# Inefficacy of Immunosuppressive Therapy for Severe Aplastic Anemia Progressing From Non-SAA: Improved Outcome After Allogeneic Hematopoietic Stem Cell Transplantation

**DOI:** 10.3389/fonc.2021.739561

**Published:** 2021-09-21

**Authors:** Limin Liu, Xin Zhao, Miao Miao, Yanming Zhang, Wenjing Jiao, Meiqing Lei, Huifen Zhou, Qingyuan Wang, Yifeng Cai, Liyun Zhao, Xiaohui Shangguan, Zefa Liu, Jinge Xu, Fengkui Zhang, Depei Wu

**Affiliations:** ^1^National Clinical Research Center for Hematologic Diseases, Jiangsu Institute of Hematology, The First Affiliated Hospital of Soochow University, Suzhou, China; ^2^State Key Laboratory of Experimental Hematology, National Clinical Research Center for Blood Diseases, Anemia Therapeutic Center, Institute of Hematology & Blood Diseases Hospital, Chinese Academy of Medical Sciences & Peking Union Medical College, Tianjin, China; ^3^Department of Hematology, The Affiliated Huai’an Hospital of Xuzhou Medical University and The Second People’s Hospital of Huai’an, Huai’an, China; ^4^Department of Hematology, Xian Yang Central Hospital, Xianyang, China; ^5^Department of Hematology in Haikou Municipal People’s Hospital, Affiliated Haikou Hospital Xiangya School of Medicine Central South University, Haikou, China; ^6^Department of Hematology, The Affiliated Hospital of Nantong University, Nantong, China; ^7^Department of Hematology, People Hospital of Xingtai, Xingtai, China; ^8^Department of Hematology, Longyan First Hospital, Affiliated to Fujian Medical University, Longyan, China; ^9^Department of Hematology, People Hospital of Xinghua, Xinghua, China; ^10^Department of Hematology, The Second Affiliated Hospital of Xuzhou Medical University, Xuzhou, China

**Keywords:** severe aplastic anemia, immunosuppressive therapy, hematopoietic stem cell transplantation, first-line therapy, outcome

## Abstract

**Background and Aims:**

This study aimed at comparing the efficacy and safety of severe aplastic anemia (SAA) cases that had met the criteria for SAA at the time of diagnosis (group A) with SAA that had progressed from non-SAA (NSAA) (group B), both undergoing first-line immunosuppressive therapy (IST). Additionally, group B was compared with SAA that had progressed from NSAA and who had been treated by allogeneic hematopoietic stem cell transplantation (allo-HSCT) (group C).

**Methods:**

We retrospectively compared 608 consecutive patients in group A (n = 232), group B (n = 229) and group C (n = 147) between June 2002 and December 2019. Six months after treatment, the rate of overall response and the fraction of patients who had achieved normal blood values, treatment-related mortality (TRM), secondary clonal disease, 5-year overall survival (OS) and failure-free survival (FFS) were indirectly compared between group A and group B, group B and group C.

**Results:**

Six months after treatment, the rate of overall response and the fraction of patients who had achieved normal blood values in group A was higher than in group B (65.24% *vs*. 40.54%, *P* < 0.0001; 23.33% *vs*. 2.25%, *P* < 0.0001); the same was true for group C (92.50% *vs*. 2.25%, *P* < 0.0001). The rate of relapse in group B was higher than in group C (*P* < 0.0001), but there were no differences in TRM and secondary clonal disease (*P* > 0.05). There were no differences in estimated 5-year OS between groups A and B (83.8% ± 2.6% *vs*. 85.8% ± 2.6%, *P* = 0.837), or between B and C (85.8% ± 2.6% *vs*. 77.9% ± 3.4%, *P* = 0.051). The estimated 5-year FFS in groups A and C was higher than for group B (57.1% ± 3.3% *vs*. 39.7% ± 3.4%, *P* < 0.001; 76.7% ± 3.5% vs. 39.7% ± 3.4%, *P* < 0.0001).

**Conclusion:**

These results indicate that IST is less effective in SAA progressing from non-SAA but allo-HSCT can improve outcomes.

## Introduction

Severe aplastic anemia (SAA) is a bone marrow (BM) failure disorder characterized by pancytopenia, and high mortality when untreated (1, 2). Allogeneic hematopoietic stem cell transplantation (allo-HSCT) from a matched related donor (MRD) is the initial treatment of choice for newly-diagnosed SAA or very SAA (vSAA) patients less than 35 years of age, HSCT may be considered, using a MRD or a suitably matched unrelated donor (MUD) if no MRD is available, for patients aged 35–50 (3). Unfortunately, less than 30% of patients who require allo-HSCT have human leukocyte antigen (HLA)-matched siblings (4). According to current therapeutic algorithms, immunosuppressive therapy (IST) with a combination of antithymocyte globulin (ATG) and cyclosporin A (CsA) is the preferred first-line treatment for patients without an MRD, and also for older patients (3, 5). The hematologic response to IST with a combination of ATG and CsA has remained steady at 60%–70% of patients over the years (6). In general, 80–90% of responders survive long-term, but for non-responders, long-term survival is only 20%–30% (7). The addition of a third drug to the IST regimen has not been beneficial (8–11), and the use of more-potent lymphocytotoxic agents has resulted in conflicting results (12, 13). Thus, how to increase the hematologic response rate is a challenge for the management of patients with SAA.

The paucity of available donors for HSCT encourages research on alternative sources of stem cells. For pediatric patients, up-front MUD has been shown to yield 96% long-term survival, similar to up-front MRD-HSCT (14). For this reason, patients younger than 20 years without an MRD have the option to proceed to an MUD-HSCT as first-line therapy (2). In addition, haploidentical family donor HSCT (HID-HSCT) offers donor availability without delay. The efficacy of HID-HSCT using post-transplant CY for SAA patients has been demonstrated by the Johns Hopkins group (15, 16). HID-HSCT as a treatment for SAA has greatly improved over the years (17–22). Patients who underwent HID-HSCT as an initial therapy had similar primary engraftment and survival outcomes to SAA patients who received MRD-HSCT (23). In China, alternative donor HSCT is recommended for newly-diagnosed young SAA patients without an MRD (24).

For non-severe aplastic anemia (NSAA) not dependent on transfusions, with blood counts within the normal range, it is reasonable to monitor the patient regularly without initially instigating immunosuppressive therapy (25). The clinical course of transfusion-independent NSAA can be variable; it can resolve spontaneously, persist independently of transfusion, or progress to transfusion-dependent NSAA or SAA (26). For NSAA, ATG + CsA resulted in significantly higher response rates and better event-free survival than CsA alone (25). For SAA patients, certain factors predict a good response to IST, such as young age, less severe disease, absolute reticulocyte count >25 ×10^9^/l and absolute lymphocyte count >1.0×10^9^/l, and the chromosomal abnormalities trisomy 8 or del(13q) (3). The absence of paroxysmal nocturnal hemoglobinuria (PNH) and shorter telomere length as independent unfavorable predictors of IST response has also been reported (27). However, very few clinical trials have been conducted for patients with SAA that has progressed from NSAA. At present, we do not know whether there are any differences between IST outcomes for newly-diagnosed SAA relative to SAA that has progressed from non-SAA. In the present multicenter study, we retrospectively compared SAA that had progressed from NSAA in patients undergoing first-line IST (n=229) with patients meeting the criteria for SAA at the time of diagnosis (n=232), as well as with 147 patients that had progressed from NSAA to SAA and who had been treated by allo-HSCT between June 2002 and December 2019.

## Patients and Methods

### Patients

Between June 2002 and December 2019, 608 consecutive patients were analyzed as follows: (i) SAA patients who had met the criteria for SAA at the time of diagnosis (group A) and underwent first-line IST or (ii) SAA progressing from NSAA in patients who underwent first-line IST (group B) and (iii) patients with SAA progressing from NSAA who underwent first-line allo-HSCT (group C) at our centers. Patients met the following criteria: diagnosis and management of SAA or vSAA, as defined by the guidelines (3); allo-HSCT or IST (ATG/antilymphocyte immunoglobulin [ALG] plus CsA) as an initial treatment; transfusion dependence; voluntary participation in allo-HSCT or IST; and absence of severe liver, kidney, lung, or heart disease. None of the patients enrolled in this study had a history of viral infection or exposure to drugs or other toxic agents. The exclusion criteria were the following: patients who were pregnant, who were diagnosed with other immunological diseases, or who tested positive for myelodysplastic syndrome (MDS) based on bone marrow analyses. Fanconi anemia was excluded by the mitomycin C-induced chromosomal breakage test. In all patients, BM cytogenetic analyses were performed and PNH screening was performed by flow cytometry using anti-CD55 and anti-CD59 antibodies. All patients and donors provided written informed consent for this protocol. This study was approved by our hospital’s Ethics Committee.

### IST Treatment Protocol

Kidney and liver function as well as infectious foci were examined to exclude contraindications for therapy before treatment. All patients were treated in sterile rooms with strict reverse isolation from the beginning of therapy. The patients in the IST group were treated with rabbit ATG (rATG) 3.75 mg/kg/day or porcine antihuman lymphocyte immunoglobulin (pALG) 30 mg/kg/day from day 1 to day 5 (28). Oral CsA treatment (3–5 mg/kg/day) was started on the first day of rATG/pALG, and was administered for at least 2 years (with dose adjustments to achieve a whole-blood trough level of 200–250 ng/ml for adults and 150–200 ng/ml for children). All patients were treated with 5 μg/kg/day G-CSF SQ from day 8. When the neutrophil count was ≥ 2.0 × 10^9^/l the dose of G-CSF was decreased or stopped. During the immunosuppressive period in accordance with institutional guidelines, prophylactic antibiotics and antifungal (voriconazole 200 mg/12 h PO or micafungin 300 mg/day i.v.) and antiviral (acyclovir 200 mg/8 h PO) agents were administered.

### HLA Typing and Donor Selection

HLA-A, -B, -C, DRB1 and -DQB1 typing of the recipients was performed. Donors were selected based on HLA typing, age, gender, health conditions and willingness to donate. MRDs were the first-choice treatment. When an MRD was unavailable, matched unrelated donors (MUDs) or haploidentical donors (HIDs) were selected.

### Treatment Protocol for Allo-HSCT

Before transplantation, when the serum ferritin level was > 1000 μg/l, iron chelation therapy was administered so that this was < 1000 μg/l. The days before transplantation are preceded by “−” and the days after the last stem cell infusion are preceded by “+”. Patients with an MRD were conditioned with the FLU/CY-based regimen, which consisted of the following: Fludarabine (FLU): 30 mg/m^2^/day intravenously (i.v.) on day −7 to −2; Cyclophosphamide (CY): 50 mg/kg/day i.v. on day −4 to −3; and antithymocyte immunoglobulin (ATG, rabbit, Thymoglobuline^®^, Genzyme, Cambridge, MA, USA): 2.5 mg/kg/day i.v. on day −8 to −4 or antilymphocyte immunoglobulin (ALG, porcine, Wuhan Institute of Biological Products, China): 20 mg/kg/day i.v. on day −8 to −4 or anti-thymocyte globulin-Fresenius (ATG-F): 5 mg/kg/day i.v. on day −8 to −4. Patients with HIDs were treated with the BU/CY-based regimen, which consisted of the following: Busulfan (Bu): 3.2 mg/kg/day i.v. on day −7 and −6; CY: 50 mg/kg/day i.v. on day −5 to −2; and ATG: 2.5 mg/kg/day i.v. on day −5 to −2 or ALG: 20 mg/kg/day i.v. on day −5 to −2 or anti-thymocyte globulin-Fresenius (ATG-F): 5 mg/kg/day i.v. on day −5 to −2. Patients with MUD were treated with the FLU/CY-based or BU/CY-based regimen.

Details of stem cell mobilization, graft collection and infusion, graft *versus* host disease (GVHD) prophylaxis and treatment strategy, supportive care, and post-transplantation surveillance were as in our previous reports (22, 29). The first day of stem cell infusion was designated “day 01” and the second day of infusion “day 02”. On day 01, BM grafts were collected by BM aspiration in an operating room and the peripheral blood stem cells (PBSCs) were collected on day 02. Fresh and unmanipulated BM and PBSCs were infused into the recipient on the day of their collection.

### Definitions

Complete response (CR) was defined as transfusion independence associated with a hemoglobin level in the normal range for age, a neutrophil count of > 1.5 × 10^9^/l, and a platelet count of > 150 × 10^9^/l. Partial response (PR) was defined as no longer meeting the criteria for SAA and no dependence on transfusion for platelets or red blood cells. Transfusion dependence was classified as no response (NR) (3). Patients who died within 90 days of IST and patients who underwent HSCT prior to 6 months from the start of therapy were considered as non-responders (3, 30). Relapse was defined as the recurrence of disease.

After HSCT, the first time that the absolute neutrophil count (ANC) exceeded 0.5 × 10^9^/l for three consecutive days was defined as neutrophil engraftment. The first time the platelet count of > 20 × 10^9^/l without transfusion support for seven consecutive days was defined as platelet engraftment. Primary graft failure was defined as failure to achieve neutrophil engraftment after HSCT by day +28. Secondary graft failure was defined as the absence of graft function after achievement of initial full engraftment (31). Delayed platelet recovery was defined as platelet engraftment only after > 30 days (3).

Death without disease progression was defined as treatment-related mortality (TRM). Early mortality was defined as death within the first 60 days after HSCT or IST treatment. Failure-free survival (FFS) was defined as survival with a therapeutic response. Death, no response by 6 months, disease progression requiring clinical intervention, or relapse were considered treatment failures for IST. Death, engraftment failure, graft rejection, or relapse were considered treatment failures for HSCT.

### Statistical Analysis

Statistical analyses were conducted on the basis of data available from the date of treatment to the final date of patient follow-up (November 30, 2020). Patient characteristics were compared using the chi-square test and non-parametric test for continuous variables. Cumulative incidence of GVHD was estimated using the competing risk model, with death as the competing event. The probabilities of OS and FFS were estimated from the time of treatment initiation using the Kaplan–Meier method and compared between different patient groups using the log-rank test. For multivariate analyses, the Cox proportional hazard regression model was used to analyze OS and FFS. Statistical analyses were performed with SPSS version 16.0 (SPSS, Chicago, IL, U.S.). All *P* values are two-sided, and results were considered statistically significant when *P* < 0.05.

## Results

### Patient Characteristics

A total of 608 patients were enrolled in this study. Of these, 232 (38.16%) were in group A, 229 (37.66%) in group B and 147 (24.18%) in group C. The clinical data included data from 128 male patients and 104 female patients in group A, 131 male patients and 98 female patients in group B and 102 male patients and 45 female patients in group C; data from 73 vSAA patients in group A, 31 vSAA patients in group B and 21 vSAA patients in group C were included. The median age was 23 years (range 1–81) in group A, 23 years (range 1–74) in group B, 24 years (range 1–58) in group C. Six (2.59%) patients in group A, 11 (4.80%) patients in group B and 17 (11.56%) patients in group C had paroxysmal nocturnal hemoglobinuria (PNH) clones. The median interval from SAA diagnosis to stem cell transplantation was 1.0 months (range 0.2–15.0) in group A, 36.0 months (range 0.5–480.0) in group B and 37.0 months (range 1.8–360.0) in group C. Characteristics of patients and donors of allo-HSCT are shown in **Tables 1** and **2**. In group B, the frequency of patients aged ≥ 40 years or who were female was higher than in group C (*P* = 0.039, *P* = 0.018). In group A, the frequency of vSAA was higher than in group B (*P* < 0.0001). In group C, the proportion of patients with a PNH clone was higher than in group B (*P* = 0.015). The median interval between diagnosis and treatment was longer in group B than A (*P* < 0.0001).

**Table 1:** Characteristics of SAA patients and clinical outcomes after treatment.

Variable	Group A (n = 232)	Group B(n=229)	Group C (n = 147)	P (Group A *vs*. Group B	P (Group B *vs*. Group C
Clinical characteristics					
Median age, years (range)	23 (1–81)	23 (1–74)	24 (1–58)	0.530	0.402
≤20 years, no. (%)	102 (43.97)	107 (46.72)	56 (38.10)	0.552	0.099
21–39 years, no. (%)	77 (33.19)	55 (24.02)	62 (42.18)	0.029	<0.001
≥40 years, no. (%)	53 (22.84)	67 (29.26)	29 (19.73)	0.117	0.039
Gender (male/female)	128/104	131/98	102/45	0.660	0.018
Disease and status at therapy, no. (%)				<0.0001	0.837
SAA	159 (68.53)	198 (86.46)	126 (85.71)		
VSAA	73 (31.47)	31 (13.54)	21 (14.29)		
SAA with PNH clone, no. (%)	6 (2.59)	11 (4.80)	17 (11.56)	0.207	0.015
Median time from diagnosis to treatment, months (range)	1 (0.2–15.0)	36 (0.5–480)	37 (1.8–360)	<0.0001	0.018
Response at 3 months, no. (%)					
CR	13 (6.07)	2 (0.88)	–	0.003	–
≤20 years, no. (%)	7 (7.07)	0 (0.00)	–	0.016	–
21–39 years, no. (%)	3 (4.35)	1 (1.85)	–	0.793	–
≥40 years, no. (%)	3 (6.52)	1 (1.54)	–	0.384	–
PR	92 (42.99)	65 (28.76)	–	0.002	–
≤20 years, no. (%)	41 (41.41)	43 (40.19)	–	0.858	–
21–39 years, no. (%)	33 (47.83)	10 (18.52)	–	0.001	–
≥40 years, no. (%)	18 (39.13)	12 (18.46)	–	0.016	–
OR	105 (49.07)	67 (29.65)	–	<0.0001	–
≤20 years, no. (%)	48 (48.48)	43 (40.19)	–	0.231	–
21–39 years, no. (%)	36 (52.17)	11 (20.37)	–	<0.001	–
≥40 years, no. (%)	21 (45.65)	13 (20.00)	–	0.004	–
Response at 6 months, no. (%)					
CR	49 (23.33)	5 (2.25)	–	<0.0001	–
≤20 years, no. (%)	23 (23.23)	2 (1.87)	–	<0.0001	–
21–39 years, no. (%)	19 (28.36)	1 (1.89)	–	<0.0001	–
≥40 years, no. (%)	7 (15.91)	2 (3.23)	–	0.051	–
PR	88 (41.90)	85 (38.29)	–	0.443	–
≤20 years, no. (%)	39 (39.39)	56 (52.34)	–	0.063	–
21–39 years, no. (%)	29 (43.28)	15 (28.30)	–	0.091	–
≥40 years, no. (%)	20 (45.45)	14 (22.58)	–	0.013	–
OR	137 (65.24)	90 (40.54)	–	<0.0001	–
≤20 years, no. (%)	62 (62.63)	58 (54.21)	–	0.221	–
21–39 years, no. (%)	48 (71.64)	16 (30.19)	–	<0.0001	–
≥40 years, no. (%)	27 (61.36)	16 (25.81)	–	<0.001	–
Patients with normal blood routine at 6 months, no. (%)	49 (23.33)	5 (2.25)	111 (92.50)	<0.0001	<0.0001
Early death, no. (%)	17 (7.33)	2 (0.87)	15 (10.20)	<0.001	<0.0001
Relapse, no. (%)	17 (13.46)	14 (16.67)	0 (0.00)	0.525	<0.0001
Secondary clonal disease, no. (%)	6 (2.59)	4 (1.75)	0 (0.00)	0.765	0.273
Median follow-up time among living patients, months (range)	60 (7–199)	50 (7–160)	54 (5–223)	0.005	0.433

SAA, severe aplastic anemia; VSAA, very severe aplastic anemia; PNH, paroxysmal nocturnal hemoglobinuria; CR, complete response; PR, partial response; OR, overall response.

**Table 2:** Characteristics of SAA patients and donors in allo-HSCT group.

Variable	N = 147
Donor median age, years (range)	30 (9–63)
Type of donor, no (%)	
MRD	50 (34.01)
UD	25 (17.01)
Haploidentical	72 (48.98)
Donor–recipient sex match, no. (%)	
Male–male	65 (44.22)
Male–female	33 (22.45)
Female–male	37 (25.17)
Female–female	12 (8.16)
Donor–recipient relationship, no. (%)	
Mother–child	9 (6.12)
Father–child	30 (20.41)
Child–mother	5 (3.40)
Child–father	10 (6.80)
Siblings	68 (46.26)
Unrelated donor	25 (17.01)
Blood types of donor to recipient, no. (%)	
Matched	77 (52.38)
Major mismatched	24 (16.33)
Minor mismatched	32 (21.77)
Major and minor mismatched	14 (9.52)
Conditioning regimen, no. (%)	
FLU/CY based	56 (38.10)
BU/CY based	91 (61.90)
Source of graft, no. (%)	
BM	9 (6.12)
PB	45 (30.61)
BM + PB	93 (63.27)
HLA-matched, no. (%)	
5/10	74 (50.34)
7/10	2 (1.36)
8/10	2 (1.36)
9/10	5 (3.40)
10/10	64 (43.54)
Median MNC, ×10^8^/kg (range)	10.76 (2.33–33.40)
Median CD34^+^ cells, ×10^6^/kg (range)	4.07 (0.68–12.39)
Median neutrophil recovery, days (range)	11 (8–37)
Median platelet recovery, days (range)	14 (8–330)
Primary graft failure, no. (%)	1 (0.72)
Secondary graft failure, no. (%)	4 (2.90)
Graft failure of platelets, no. (%)	5 (3.62)
Delayed platelet recovery, no. (%)	15 (10.87)

SAA, severe aplastic anemia; allo-HSCT, allogeneic hematopoietic stem cell transplantation; MRD, matched related donor; UD, unrelated donor; FLU, Fludarabine; CY, Cyclophosphoramide; BU, Busulfan; BM, bone marrow; PB, peripheral blood; MNC, mononuclear cell.

### Outcomes of IST

Three months after treatment, 18 patients in group A had died, leaving 214 evaluable patients; in group B two patients died, so there were 227 evaluable patients. The rate of CR in group A and B was 6.07% and 0.88%, respectively (*P =* 0.003); the frequency of PR was 42.99% and 28.76%, respectively (*P =* 0.002); and the rate of OR was 49.07% and 29.65%, respectively (*P* < 0.0001). In patients ≤ 20 years of age, there was no difference in the OR rate between groups A and B (48.48% *vs*. 40.19%, *P* = 0.231), but in patients aged 21–39 years and ≥ 40 years, OR was more frequent in group A than group B (52.17% *vs*. 20.37%, *P* < 0.001; 45.65% *vs*. 20.00%, *P* = 0.004). In group A, the 22 patients responses to IST at 6 months were not evaluable because of death (*n* = 20) or because the patient received HSCT from an alternative donor within 6 months (*n* = 2). Thus, 210 were evaluable. In group B, 7 patients died, leaving 222 evaluable patients. The CR rate in groups A and B was 23.33% and 2.25%, respectively (*P* < 0.0001); the PR rate was 41.90% and 38.29%, respectively (*P* = 0.443); and the OR rate was 65.24% and 40.54%, respectively (*P* < 0.0001). In patients ≤ 20 years of age, there was no difference in the OR rate between groups A and B (62.63% *vs*. 54.21%, *P* = 0.221); in 21–39 year-old and ≥ 40 year-old patients, the OR rates in group A were higher than in group B (71.64% *vs*. 30.19%, *P* < 0.0001; 61.36% *vs*. 25.81%, *P* < 0.001). (**Table 1**).

During follow-up, 24 cases in group A were NR at 6 months, and therefore received salvage HSCT from an MRD (*n* = 4), a haploidentical family donor (*n* = 16), an unrelated cord blood donor (*n* = 1) or an MUD (*n* = 3); In group B, 21 non-responders received salvage HSCT from an MRD (*n* = 3), a HID (*n* = 10), or an MUD (*n* = 8).

### Outcomes of Allo-HSCT

In the allo-HSCT group (group C), 138 of the 147 patients survived for more than 28 days; the median dose of mononuclear cells in the grafts was 11.61 (2.33–33.40) × 10^8^/kg, and the CD34^+^ cell dose was 4.04 (0.68–12.39) × 10^6^/kg. Fifteen patients suffered early death. Among the 138 evaluable patients, one experienced primary graft failure, but all the others achieved successful engraftment. However, 4 patients experienced secondary GF, and 5 suffered platelet GF, with another 15 experiencing delayed platelet engraftment. The median time to neutrophil engraftment was 11 days (range, 8–37) and to platelet engraftment 14 days (range, 8–330) (**Table 2**). Six months after allo-HSCT, 111 of the 120 evaluable patients had achieved normal blood counts, very much higher than in group B (92.50% *vs*. 2.25%, *P* < 0.0001) (**Table 1**).

The cumulative incidence of acute GVHD (aGVHD) grades II to IV on day +100 was 14.18%, and of grades III to IV aGVHD 5.71%. The cumulative incidence of chronic GVHD (cGVHD) was 21.18%, with moderate–severe cGVHD at 6.97%.

### Treatment-Related Mortality, Relapse, and Secondary Clonal Disease

**Figure 1:**
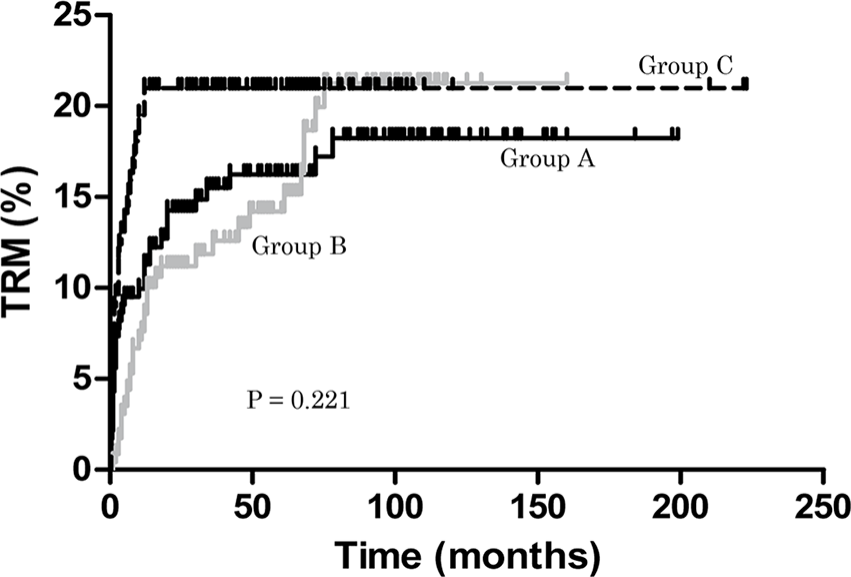
Treatment-related mortality (TRM). During the follow-up period, TRM in groups A, B and C was 18.25%, 21.25% and 20.98%, respectively (*P =* 0.221).

During the follow-up period, TRM in groups A, B and C was 18.25%, 21.25% and 20.98%, respectively (*P =* 0.221) (**Figure 1**). None of the patients in group C relapsed, but 17 (13.46%) and 14 (16.67%) responders relapsed in groups A and B, respectively. This was significant for group B compared with C (*P* < 0.0001). In group C, no patient developed secondary clonal disease, but in group A 6 (2.59%) patients did so (three PNH, three MDS) and in group B 4 (1.75%) patients developed secondary clonal disease (four MDS). However, there were no differences in the rate of secondary clonal disease (P > 0.05) (**Table 1**). In group B, one patient developed diffuse large B-cell lymphoma (DLBCL) one year after treatment.

### Survival

**Figure 2:**
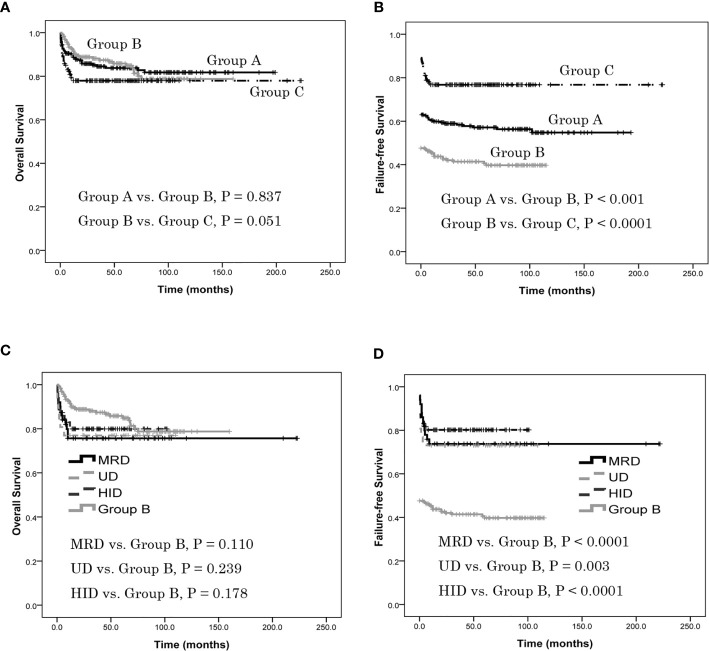
Patients overall survival (OS) and failure-free survival (FFS), as assessed using Kaplan-Meier analysis. **(A)** The estimated OS at 5 years was 83.8% ± 2.6%, 85.8% ± 2.6% and 77.9% ± 3.4% in groups A, B and C, respectively. There was no difference in estimated 5-year OS between group A and group B (*P* = 0.837), or between B and C (*P* = 0.051). **(B)** The estimated FFS at 5 years was 57.1% ± 3.3%, 39.7% ± 3.4% and 76.7% ± 3.5% in groups A, B and C, respectively. The estimated FFS at 5 years of patients in group A was higher than in group B (*P* < 0.001) as was group C (*P* < 0.0001). **(C)** In group C, the estimated 5-year OS of patients with MRD was 75.6% ± 6.1%, no different from group B (P = 0.110); the estimated 5-year OS of patients with UD was 76.9% ± 8.3%, no different from group B (P = 0.239); the estimated 5-year OS of patients with HID was 80.5% ± 4.7%, again not different from group B (P = 0.178). There was no difference of estimated 5-year OS in patients with MRD, UD or HID (P = 0.866). **(D)** In group C, the estimated FFS at 5 years of patients with MRD was 73.7% ± 6.3%, which was higher than in group B (P < 0.0001); the estimated FFS at 5 years of patients with UD was 73.1% ± 8.7%, which was higher than in group B (P = 0.003); the estimated 5-year FFS of patients with HID was 80.2% ± 4.7%, which was also higher than group B (P < 0.0001). There was no difference of estimated 5-year FFS in patients with MRD, UD or HID (P = 0.665).

The estimated OS at 5 years was 83.8% ± 2.6%, 85.8% ± 2.6% and 77.9% ± 3.4% in groups A, B and C, respectively. There was no difference in estimated 5-year OS between group A and group B (*P* = 0.837), or between B and C (*P* = 0.051) (**Figure 2A**). The estimated FFS at 5 years was 57.1% ± 3.3%, 39.7% ± 3.4% and 76.7% ± 3.5% in groups A, B and C, respectively. The estimated FFS at 5 years of patients in group A was higher than in group B (*P* < 0.001) as was group C (*P* < 0.0001) (**Figure 2B**). In group C, the estimated 5-year OS of patients with MRD was 75.6% ± 6.1%, no different from group B (*P* = 0.110); the estimated 5-year OS of patients with UD was 76.9% ± 8.3%, no different from group B (*P* = 0.239); similarly, the estimated 5-year OS of patients with HID was 80.5% ± 4.7%, again not different from group B (*P* = 0.178) (**Figure 2C**). In group C, the estimated FFS at 5 years of patients with MRD was 73.7% ± 6.3%, which was higher than in group B (*P* < 0.0001); the estimated FFS at 5 years of patients with UD was 73.1% ± 8.7%, which was higher than in group B (*P =* 0.003); the estimated 5-year FFS of patients with HID was 80.2% ± 4.7%, which was also higher than group B (*P* < 0.0001) (**Figure 2D**). There was no difference of estimated 5-year OS or FFS in patients with MRD, UD or HID in group C (*P* = 0.866, 0.665).

Considering young age, with PNH clone and time from diagnosis to treatment might affect the treatment results. In the multivariate analysis, young age was the favorable factor for OS and FFS (*P* < 0.0001, = 0.006), SAA was the favorable factor for OS (*P =* 0.001) compared with vSAA, cases that had met the criteria for SAA at the time of diagnosis was the favorable factor for FFS (*P =* 0.025), but with PNH clone and time from diagnosis to treatment did not affect OS and FFS (*P* = 0.634, 0.800, 0.583, 0.607) between group A and group B. Young age was the favorable factor for OS and FFS (*P* < 0.0001, = 0.001), HSCT was the favorable factor for FFS (*P* < 0.0001), with PNH clone and time from diagnosis to treatment did not affect OS and FFS (*P* = 0.684, 0.943, 0.768, 0.987) between group B and group C.

**Figure 3:**
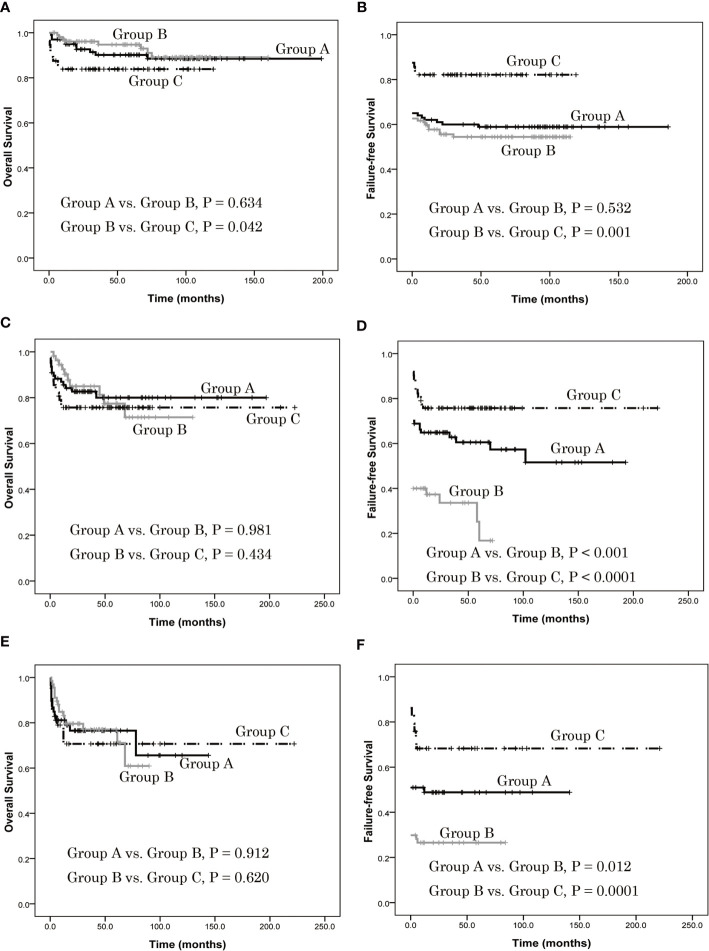
Overall survival (OS) and failure-free survival (FFS) of patients with different age group, as assessed using Kaplan-Meier analysis. **(A)** In patients ≤ 20 years of age, there was no difference in estimated 5-year OS between group A and group B (90.2% ± 3.1% *vs*. 94.7% ± 2.3%, *P* = 0.634), but it was higher in group B than in group C (94.7% ± 2.3% *vs*. 83.9% ± 4.9%, *P* = 0.042). **(B)** There was no difference in estimated 5-year FFS between group A and group B (58.4% ± 4.9% *vs*. 54.4% ± 4.9%, *P* = 0.532), but it was lower in group B than in group C (54.4% ± 4.9% *vs*. 82.1% ± 5.1%, *P* = 0.001). **(C)** In patients 21–39 years of age, there was no difference of estimated 5-year OS between groups A and B (80.0% ± 5.0% *vs*. 77.4% ± 7.1%, *P* = 0.987), or between B and C (77.4% ± 7.1% *vs*. 75.7% ± 5.5%, *P* = 0.434). **(D)** The estimated 5-year FFS of group A was higher than group B (60.5% ± 5.9% *vs*. 16.8% ± 9.1%, *P* < 0.001) as was group C (75.7% ± 5.5% *vs*. 16.8% ± 9.1%, *P* < 0.0001). **(E)** In patients ≥ 40 years old, there was no difference of estimated 5-year OS between groups A and B (76.5% ± 6.0% *vs*. 77.0% ± 5.5%, *P* = 0.912), or between groups B and C (77.0% ± 5.5% *vs*. 70.7% ± 8.8%, *P* = 0.620). **(F)** The estimated 5-year FFS of group A was higher than group B (48.8% ± 6.9% *vs*. 26.5% ± 5.4%, *P* = 0.012) as was group C (68.3% ± 8.8% *vs*. 26.5% ± 5.4%, *P* = 0.0001).

In patients ≤ 20 years of age, there was no difference in estimated 5-year OS between group A and group B (90.2% ± 3.1% *vs*. 94.7% ± 2.3%, *P* = 0.634), but it was higher in group B than in group C (94.7% ± 2.3% *vs*. 83.9% ± 4.9%, *P* = 0.042) (**Figure 3A**). Also, there was no difference in estimated 5-year FFS between group A and group B (58.4% ± 4.9% *vs*. 54.4% ± 4.9%, *P* = 0.532), but it was lower in group B than in group C (54.4% ± 4.9% *vs*. 82.1% ± 5.1%, *P* = 0.001) (**Figure 3B**). In patients 21–39 years of age, there was no difference of estimated 5-year OS between groups A and B (80.0% ± 5.0% *vs*. 77.4% ± 7.1%, *P* = 0.987), or between B and C (77.4% ± 7.1% *vs*. 75.7% ± 5.5%, *P* = 0.434) (**Figure 3C**). In contrast, the estimated 5-year FFS of group A was higher than group B (60.5% ± 5.9% *vs*. 16.8% ± 9.1%, *P* < 0.001) as was group C (75.7% ± 5.5% *vs*. 16.8% ± 9.1%, *P* < 0.0001) (**Figure 3D**). In patients ≥ 40 years old, there was no difference of estimated 5-year OS between groups A and B (76.5% ± 6.0% *vs*. 77.0% ± 5.5%, *P* = 0.912), or between groups B and C (77.0% ± 5.5% *vs*. 70.7% ± 8.8%, *P* = 0.620) (**Figure 3E**). Finally, the estimated 5-year FFS of group A was higher than group B (48.8% ± 6.9% *vs*. 26.5% ± 5.4%, *P* = 0.012) as was group C (68.3% ± 8.8% *vs*. 26.5% ± 5.4%, *P* = 0.0001) (**Figure 3F**).

## Discussion

According to current therapeutic algorithms, allo-HSCT from an MRD is the initial treatment of choice for newly-diagnosed young patients with SAA or vSAA, whereas IST with a combination of ATG/ALG and CsA is the preferred first-line treatment for patients without an MRD and also for older patients (3, 5). For NSAA, when it has progressed to transfusion-dependent NSAA or SAA, allo-HSCT or IST is recommended (3). Today, there are still many challenges in the management of patients with SAA and the aim would be to achieve “precision therapy”. As the guidelines indicate, certain factors predict a good response to IST, such as young age, less severe disease, absolute reticulocyte count > 25 × 10^9^/l and absolute lymphocyte count > 1.0×10^9^/l, and the chromosomal abnormalities trisomy 8 or del (13q) (3). Although data indicated that ATG + CsA treatment yielded significantly higher response rates and better event-free survival than CsA alone for NSAA (25), SAA with shorter time from diagnosis to treatment had higher response rates. However, at present, we do not know the results of IST for SAA that has progressed from non-SAA. Can this subgroup (i.e. SAA progressing from NSAA) also benefit from the definition of factors predicting the superior or inferior response to IST?

In the present study, the data indicated that 3 months and 6 months hematologic response rates (OR) to IST in group A were higher than in group B (*P* < 0.0001), although there were no differences in estimated 5-year OS between these groups (*P* = 0.837). However, the estimated 5-year FFS for patients in group A was higher than in group B (*P* < 0.001). This indicates that the efficacy of IST is lower in SAA that progressed from non-SAA than in patients with SAA that had met the criteria for SAA at the time of diagnosis. The mechanism of this result was unclear, a probable explanation was the CD34^+^ cell was injured in the progression of disease and the CD34^+^ cell number was lower in SAA that progressed from non-SAA than in patients with SAA that had met the criteria for SAA at the time of diagnosis. Thus, SAA progressing from non-SAA may predict an inferior response to IST. The addition of a third drug to the combination of ATG and CsA regimen has not been beneficial (6–11), and the use of more-potent lymphocytotoxic agents has resulted in conflicting data (12, 13). Hence, improving the efficacy of treatment for patients with SAA that progressed from non-SAA remains a therapeutic challenge.

In comparison to IST, HSCT allows faster restoration of hematopoiesis and lowers the risk of clonal disease (32). However, > 70% of SAA patients who require allo-HSCT do not have an MRD. Alternative sources of stem cells, from groups including MUD, HID, and unrelated umbilical cord blood (UCB) have been recommended. Patients younger than 20 years have an option to proceed to a MUD-HSCT as first-line therapy (2), but identifying a suitable donor may be very time-consuming. HID-HSCT offers donor availability without delay. In China, an alternative donor HSCT is recommended for newly-diagnosed young SAA patients without an MRD (24). In the present study, the data demonstrated that patients with SAA progressing from non-SAA who underwent allo-HSCT experienced a better clinical course than with IST, and although OS remained similar, FFS was superior. Patients with SAA that progressed from non-SAA who underwent MRD-HSCT, UD-HSCT or HID-HSCT had better FFS than those treated with IST (*P* < 0.005). Finally, there was no difference in terms of OS or FFS between MRD-HSCT, UD-HSCT and HID-HSCT (*P* = 0.866, 0.665).

For SAA patients, OS is no longer the only parameter used to determine the optimal first-line treatment. Many patients with SAA experience poor quality of life (QoL), including severe fatigue, poor global health status, impaired functioning, dyspnea, and hemorrhage. Impairment in these QoL measures has been attributed, at least in part, to downstream effects of pancytopenia in these patients. A hematologic response is strongly correlated with long-term survival and QoL. For allo-HSCT, extensive cGVHD is the most adverse factor affecting QoL (33). In the present study, the cumulative incidence of moderate–severe cGVHD was only 7.45% ± 3.21%, and the percentage of patients achieving normal blood values in group C was higher than in group B (*P* < 0.0001). The relapse rate was also lower than in group B (*P* < 0.0001). Because IST was less effective in patients with SAA that had progressed from NSAA than in patients with SAA that had met the criteria for SAA at the time of diagnosis, exploiting allo-HSCT could result in improved outcomes. Thus, for SAA progressing from NSAA, allo-HSCT may be the first therapy choice for patients who can tolerate such aggressive treatment, and for those without an MRD, UD-HSCT and HID-HSCT can be acceptable.

In conclusion, the encouraging results observed here suggest that (i) IST had inferior efficacy in patients with SAA that progressed from NSAA relative to those with SAA that had met the criteria for SAA at the time of diagnosis, (ii) allo-HSCT can improve clinical outcome (FFS) for patients with SAA that progressed from NSAA. We wish to mention that our study is limited by its retrospective nature. Further well-designed, prospective, controlled multicenter cooperative studies are needed to validate our results and to confirm the superiority of this approach.

## Data Availability Statement

The raw data supporting the conclusions of this article will be made available by the authors, without undue reservation.

## Ethics Statement

The studies involving human participants were reviewed and approved by The First Affiliated Hospital of Soochow University. Written informed consent to participate in this study was provided by the participants’ legal guardian/next of kin.

## Author Contributions

LL, XZ, and MM, wrote the manuscript and performed the analysis. DW, and FZ, designed the protocol. All authors contributed patients, provided clinical and laboratory data, and revised and corrected the manuscript. All authors contributed to the article and approved the submitted version.

## Funding

This work was partially supported by grants from the National Key R&D Program of China (2016YFC0902800, 2017YFA0104502, and 2017ZX09304021), the Innovation Capability Development Project of Jiangsu Province (BM2015004), the Jiangsu Provincial Key Medical Center (YXZXA2016002), the Jiangsu Medical Outstanding Talents Project (JCRCA2016002), the Natural Science Foundation of Jiangsu Province (Grants No.BK20180202) and the Priority Academic Program Development of Jiangsu Higher Education Institutions (PAPD).

## Conflict of Interest

The authors declare that the research was conducted in the absence of any commercial or financial relationships that could be construed as a potential conflict of interest.

The reviewer AP declared a shared affiliation with the authors XZ, FZ, to the handling editor at the time of review.

## Publisher’s Note

All claims expressed in this article are solely those of the authors and do not necessarily represent those of their affiliated organizations, or those of the publisher, the editors and the reviewers. Any product that may be evaluated in this article, or claim that may be made by its manufacturer, is not guaranteed or endorsed by the publisher.
